# State Medicine

**Published:** 1904-06-18

**Authors:** 


### STATE MEDICINE.

### HOUSING OF THE WORKING CLASSES.

The Camberwell Borough Council[1] decided that the only feasible plan of dealing with their insanitary areas was to gradually purchase property so condemned; adapt and put it into sanitary repair by expending what in private hands would be the landlords' profits, and thus continue to let it out to the poorest class of tenants at a very low rental, and under the very best sanitary conditions. Such an enterprise was rendered possible by the discovery of a useful sub-section of the Housing Act, hitherto overlooked, which provides as follows: "The local authority may, if they think fit, contract for the purchase or lease of any lodging-houses for the working classes already or hereafter to be built and provided." To purchase cheaply the precaution is taken of concealing the identity of the Council to the vendor. Out of 850 houses in the Hollington Street area no fewer than 135 had been acquired by the Council at the end of May last. To-day the number of houses is about 200, representing some 500 tenants and a population of approximately 2,500 people. On the acquisition of a house it is at once put into a state of repair, and additional sanitary accommodation is provided, while the same tenants continue in occupation and pay no extra rent for the added comfort of their surroundings. The advantages of this system are summed up under six heads: (1) No displacement of population, even temporarily. (2) Original tenants retained at the same rents. (3) No charge on the rates, as the work shows a profit. (4) Standard of living improved and death-rate lowered. (5) Sub-letting abolished. (6) Housing of the poorest of the poor at rents which save them from seeking in-door parochial relief. Altogether 72 out of 500 tenants got a free week's rent, which is the annual bonus offered by the Council to all those who, after six months' occupation, have a clear book on the rent-day before Christmas. So far this work of improvement has not cost the ratepayers a single penny. Robinson[2] thinks that the perfect scheme for the perfect housing of the poor has yet to be devised, and that among the many obstacles to its fulfilment are the imperfect state of the Parliamentary Act which gives the power to proceed and the difficulty of inducing authorities already heavily burdened with debt to undertake any outlay which may prove financially hazardous. He divides the classes to be provided for into three groups: First, tramps, vagrants, and hawkers—birds of passage who are here to-day and gone to-morrow. There is no desire to attract such undesirables by the provision of comfortable apartments, but their presence is one of the arguments used in favour of the motion of municipal common lodging-houses. Secondly, persons temporarily resident in the town and engaged in work not of a permanent character, such as navvies, pit-sinkers, and labourers. These may be housed in common lodging-houses, but failing such provision, arrangements may be made in any tenements to be erected for rehousing purposes of single-roomed apartments at a low rate of rental. The third class consists of households where the wage-earner is making not more than 15s. or 20s. per week, and who certainly cannot afford to pay more than 2s. or 3s. per week in rent. This is the most difficult class to provide for, as a sanitary house must be provided capable of accommodating four to six people, the rent to be not more than 3s., and yet which will give a sufficient return to pay interest on the outlay on the building and land and to repay the sum borrowed in an extended period of years. It is still possible to obtain land in the outskirts of most of our large towns at a sufficiently cheap rate to allow of the erection of houses with accommodation enough for an average household of this class, and at a reasonable rental. It is unwise and unnecessary to go to an extreme in the direction of comfort. He thinks that all that is required is a roomy kitchen, a scullery containing a movable bath, and a cupboard or press downstairs; while upstairs there would be two or three bedrooms. Such a house would meet the requirements of most families of this class, and could be erected at a cost which would be repaid at a rental of about 2s. 6d. per week. Where private enterprise fails to provide houses of this class, it is quite allowable for a public body to step in and provide them. Berry,[3] dealing with the same class of tenants, states that he is trying to persuade his corporation to put up houses which can be let from 2s. 6d. to 3s. 6d. per week. A rent of 1s. or 1s. 3d. per room is as much as these poor people can pay. He has come to the conclusion that municipal authorities should and must deal with (1) their insanitary property; (2) they must provide houses at a reasonable rent for those displaced, even at the expense of the rates, in the case of the very poor. The working classes are provided for, and will be, by private enterprise. The poor will have to be provided for by the local authorities or the Poor Law Guardians. In his experience if people were turned out of houses with a rental of from 2s. 6d. to 4s. per week, and no houses were provided for them excepting at a rental of from 5s. to 6s. 6d. per week, two and more families would have to occupy one house. Phillips[4] mentions that the prevailing feature in rural districts is the dearth of cottages suitable for agricultural labourers, and that there is little prospect of the scarcity being met. As a consequence houses are allowed to be occupied, which on sanitary grounds might be condemned were there a sufficiency of accommodation elsewhere. The general cessation in the erection of cottages suitable for agricultural labourers may be attributed to the excessive cost of building, due to:—(1) The increase in prices of labour and materials during the past 20 years, and (2) restrictions of local authorities, contained in building by-laws. Further, properties condemned as unfit for human habitation in some instances become mere channels of speculation. New premises, constructed to replace them, are intended for other purposes than labourers' dwellings. Thus the Act intended for the better housing of the poor, becomes both an instrument by which the labouring classes are displaced, and a useful lever for speculative purposes. It is not a specific duty of sanitary authorities to follow occupiers who have been displaced, but it is tacitly understood in the present state of rural housing, that the only course open to the tenants is to move to some spot where they may find houses similar to those vacated, and where to get any shelter they are perforce compelled to huddle together with others. The inevitable result is overcrowding, and the dreary repetition of proceedings. The rural housing question[5] emphasises the need of country areas being placed under as efficient sanitary administration as towns. Children should be taught at school, and in their homes, the true value of good sanitation. Riley,[6] referring more particularly to overcrowding, mentions that it has been laid down in the operations of the London County Council that not less than 400 cubic feet of air-space shall be provided per person in their dwellings (for children under 10 years 200 cubic feet), and that in any tenement the total number of persons should work out at not more than two to a room, but infants born in the tenements do not count until they attain five years of age, when they are reckoned as half an adult up to the age of 10 years. The last census returns show that the population increased during the 10 years 1891 to 1901 from 4,228,317 to 4,536,541. Notwithstanding this increase of 308,224 the number of single-room tenements in which more than two persons were "enumerated" declined from 56,727 to 40,762; whilst the number of one-room tenements with six or more inmates fell from 4,097 to 1,802. With regard to the one-room tenements occupied by more than two persons, not only is there found a reduction in the number of such tenements, but more important still, a reduction in the average number of persons occupying those tenements—the number per room in 1901 being 3·62, as against 3·80 in 1891. Moreover, the number of persons living more than two in a room in tenements of one to four rooms decreased from 831,668 in 1891, to 726,096 in 1901, or from 19·66 per cent. to 16 per cent. The new workmen's dwellings[7] erected in the City of Westminster contain 793 rooms, divided into 342 tenements, accommodating about 1,600 persons, and the rents of the tenements range from 3s. to 4s. 3d. per week for one room, to 11s. 6d. to 12s. 6d. per week for four rooms. These rents are considerably under those ruling in the neighbourhood for tenements with rooms of a much smaller area and not nearly so well fittted up with cupboards and other conveniences. In order that the rents should be as low as possible, the one-, two-, and three-room tenements have been built on the "associated" principle, *i.e.* the sanitary accommodation and laundries are shared by the tenants on each floor. All the three- and four-room tenements have entrance lobbies to give greater privacy. Every room is fitted with Venetian blinds, and each living room is supplied with a closed range with removable oven. Each bedroom has been provided with stove and a good-sized cupboard for clothes. Every tenement is lighted by gas on the penny-in-the-slot system. On the landings are laundries, with boilers and glazed ware washing troughs. There is an urn room for the supply of hot water, and a drying-room, and provision is even made for cycles and perambulators. The cost of the land and the building has been approximately £95,000, and the rents have been adjusted to a scale that will, after providing for a sinking fund to repay the total outlay on the buildings in sixty years and on the land in eighty years, give a net return on the expenditure of 3¾ per cent. per annum. The dwellings are only let to members of the working classes employed at limited wages within the City of Westminster.

[1] Pub. Health Engineer, Jan. 16, 1904. [2] Pub. Health, Jan. 1904. [3] Ibid. [4] Pub. Health Eng., April 2, 1904. [5] Ibid, April 9, 1904. [6] Ibid, April 2, 1904. [7] Ibid, May 14, 1904.

